# Co-administration of ritonavir-boosted protease inhibitors and rate of tenofovir discontinuation in clinical practice

**DOI:** 10.7448/IAS.17.4.19571

**Published:** 2014-11-02

**Authors:** Silvia Costarelli, Alessandro Cozzi-Lepri, Giuseppe Lapadula, Stefano Bonora, Giordano Madeddu, Franco Maggiolo, Andrea Antinori, Andrea Gori, Antonella D'Arminio-Monforte

**Affiliations:** 1 Infectious Diseases, San Gerardo Hospital, Monza, Italy; 2 Virology, Royal Free and University College Medical School, London, UK; 3 Infectious Diseases, University of Torino, Torino, Italy; 4 Infectious Diseases, University of Sassari, Sassari, Italy; 5 Infectious Diseases, Papa Giovanni XXIII Hospital, Bergamo, Italy; 6 Infectious Diseases, Spallanzani Hospital, Rome, Italy; 7 Infectious Diseases, University of Milan, Milano, Italy

## Abstract

**Introduction:**

In clinical trials, toxicity leading to discontinuation of tenofovir (TDF) is a rare occurrence (3% by two years)[1, 2]; however, in clinical practice it seems to be higher. Previous studies suggested that TDF toxicity is higher when it is co-administered with ritonavir-boosted protease inhibitors (PI/r)[3, 4]. The aim of this study is to assess the rate of TDF discontinuations in clinical practice and to explore associated factors.

**Methods:**

All previously antiretroviral-naïve patients initiating a TDF-containing regimen were selected from the ICONA cohort, unless they were positive for hepatitis B. The primary outcome was TDF discontinuation (>30 days) regardless of the reason, the secondary was TDF discontinuation due to toxicity. All analyses were repeated for the isolated stop of TDF (no stop of associated drugs). The main reason for discontinuation as reported by the treating physicians was used to classify stops. Kaplan–Meier (KM) analysis and Cox proportional hazards model were used.

**Results:**

A total of 3,303 naïve patients were enrolled: 674 (20.4%) were female, the median age was 38 years (32–45), 55% were on PI/r-based regimen and 45% on NNRTI; 80% of calculated estimated glomerular filtration rates (eGFR) were >90 ml/min. The probability of discontinuation of TDF regardless of the reason was 10% (95% CI 8–11) at two years, 20% by eight years. The causes of discontinuation were: toxicity (33%), failure (10%), non-adherence (21%), simplification (16%) and other/unknown causes (20%). The five-year KM estimates in the PI/r vs. not PI/r groups were 23% vs. 10%, respectively (log-rank p=0.0001), for the outcome of stopping regardless of the reason, and 8% vs. 4% (p=0.18) for discontinuation due to toxicity. In a multivariable Cox model, PI/r use and lower body weight were associated with increased risk of discontinuing TDF regardless of the reason; lower eGFR at baseline was associated with TDF discontinuation for toxicity and PI/r use was associated with isolated stop of TDF (Figure). No differences in rates of TDF discontinuations between PIs were found.

**Conclusion:**

In our cohort, the observed frequency of TDF discontinuations was low although higher than estimated in clinical trials (10% by two years). Co-administration of TDF with PI/r was associated with an increased rate of TDF discontinuations. This finding should guide further investigations of the mechanism that may have led to discontinuation of TDF in patients using PI/r.

**Figure 1:**
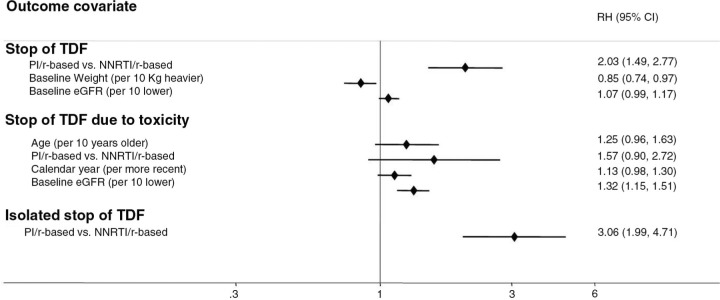
Factors associated with risk of stopping TDF. Adjusted RH from fitting a Cox regression model. Model also adjusted for: age, gender, mode of HIV transmission, HCV status, AIDS diagnosis, CD4 and VL at time of starting TDF nadir CD4 count, nationality, diabetes and use of blood pressure lowering drugs. Only factors with p<0.1 shown in graph.
